# Association of trabecular texture and paraspinal muscle characteristics with prevalent vertebral fractures - QCT results from a subcohort of the AGES population

**DOI:** 10.1186/s12891-026-09893-9

**Published:** 2026-06-27

**Authors:** Tobias Stumpf, Oliver Chaudry, Jana Hummel, Sandra Freitag-Wolf, Eren Yilmaz, Stefan Bartenschlager, Sigurdur Sigurdsson, Vilmundur Gudnason, Nicolai R. Krekiehn, Claus C. Glüer, Klaus Engelke

**Affiliations:** 1 Institute of Radiology, Friedrich-Alexander-University Erlangen-Nürnberg and Universitätsklinikum Erlangen, Erlangen, Germany; 2 Department of Medicine 3, Friedrich-Alexander-University Erlangen-Nürnberg and Universitätsklinikum Erlangen, Erlangen, Germany; 3 Institute of Medical Informatics and Statistics, Kiel University and University Hospital Schleswig-Holstein, Kiel, Germany; 4 Section Biomedical Imaging, Department of Radiology and Neuroradiology, Kiel University and University Hospital Schleswig-Holstein, Kiel, Germany; 5 Icelandic Heart Association Research Institute, Kopavogur, Iceland

**Keywords:** Fracture prediction, Incident and prevalent vertebral fracture, Computed tomography; BMD, Paraspinal muscle, Trabecular texture

## Abstract

**Background:**

Prediction of osteoporotic fractures remains suboptimal, leaving many high-risk individuals untreated while others fracture without prior diagnosis. Muscle characteristics are not part of prediction algorithms although studies showed that paraspinal muscle size, density, and fat infiltration are associated with vertebral fractures (VF). However, an analysis of the prospective AGES-Reykjavik study did not show improved prediction of the first incident VF when combining muscle parameters with BMD.

**Objective:**

To determine whether paraspinal muscle characteristics are associated with prevalent fractures using the prospective AGES-Reykjavik study.

**Methods:**

In the current study associations of muscle parameters with prevalent VF were analyzed in the same cohort of 486 women and 340 men with 96 and 78 prevalent VF, respectively. 50 parameters from CT scans of the L1 and L2 vertebrae, divided into a BMD, a trabecular texture and a muscle subset were used as discriminators. Each subset also included age and BMI. The number of parameters was reduced using stepwise logistic regression to create multivariable fracture discrimination models. Model accuracy was assessed using the likelihood ratio test (LRT) and the area under the curve (AUC). Bootstrap analyses were performed to assess stability of the model selection process.

**Results:**

23 parameters significantly discriminated prevalent VFs univariately in women and 5 in men. In women multivariable bone and muscle models showed significantly better fracture discrimination (*p* < 0.01) than the combination of age and BMI. Compared to the BMD model, LRT showed a significantly improved VF discrimination of the combinations of BMD with texture or with muscle models (*p* < 0.001). In men the BMD model (AUC 0.64) did not significantly improve VF discrimination compared to age and BMI.

**Conclusions:**

In older women, but not men, paraspinal muscle characteristics significantly enhance the discrimination of prevalent VFs beyond BMD alone. Muscle parameters were not predictive of incident VFs in prior analyses of the same cohort, suggesting that muscle deterioration may occur concurrently or follow vertebral fracture.

**Supplementary Information:**

The online version contains supplementary material available at 10.1186/s12891-026-09893-9.

## Background

Reliable fracture risk prediction plays a central role in clinical decision-making for osteoporosis management. Fracture risk is most commonly estimated using a composite approach that integrates age with clinical risk factors. Bone mineral density (BMD) and the presence of morphologic vertebral fractures are also well-established contributors to fracture risk; however, their assessment necessitates additional imaging procedures such as spinal radiography, dual-energy X-ray absorptiometry (DXA), and computed tomography (CT). Despite these measures, current fracture prediction tools such as FRAX, QFracture, or the Garvan or the German DVO fracture risk calculators demonstrate only moderate sensitivity and specificity [1, 2]. Ultimately, current pharmacological therapies prevent fractures in fewer than 30% of treated patients [3]. Evidence also suggests that the majority of fractures occur in individuals with non-osteoporotic T-scores who are rarely identified for treatment [4]. These limitations have driven the development of alternative strategies aimed at improving fracture risk assessment. Such approaches include fall-risk evaluation and gait analysis [5] finite element analysis (FEA) to determine bone strength [6, 7] and quantitative assessment of trabecular bone texture [8, 9]. In recent years, increasing attention has also been directed toward the contribution of muscle characteristics.

The interaction between bone and muscle, initially conceptualized in Frost’s mechanostat theory in 1996 [10] have since been extensively explored [11–14]. Several studies have demonstrated associations between vertebral fractures and paraspinal muscle size [15–26], muscle density [18, 22, 27, 28] and degree of fatty infiltration [16, 17, 20, 23, 25, 29]. A meta-analysis encompassing 11 studies published before 2023 further confirmed a significant association between paraspinal muscle parameters and vertebral fractures [30]. With the exception of one study [22], however, most studies evaluated muscle characteristics either in relation to prevalent vertebral fractures or secondary incident fractures [24, 28, 31, 32]. Among the two prospective studies, one large Chinese cohort study [22, 33] found that erector spinae muscle area and density were significant predictors of incident vertebral fractures in women, while muscle area alone was predictive in men, after adjustment for age, BMI, and sex. In contrast, the prospective AGES-Reykjavik study reported differing results [34], showing that parameters of muscle fat infiltration predicted the first incident vertebral fracture in men but not in women. Furthermore, multivariable analysis indicated that even in men, muscle density was no longer a significant predictor once lumbar spine BMD was included in the model.

These findings suggest differences between the associations of muscle parameters with prevalent vertebral fractures and their utility in predicting incident fractures. However, it is difficult to define these differences precisely due to substantial heterogeneity across studies. Variations include differences in ethnicity and age of study populations, the use of MRI versus CT imaging, differences in anatomical coverage, limited consistency in image processing methods and muscle parameters assessed, and wide variability in statistical approaches, from simple T-score comparisons to complex multivariable models. Sex-specific analyses and adjustments for BMD were also infrequently performed.

Accordingly, the present study builds on a prior analysis [34] that compared the prognostic value of multiple CT-based measures, including BMD, trabecular texture, and paraspinal muscle characteristics, using both univariate and multivariable models. That analysis utilized a subset of data from the AGES-Reykjavik study, a large epidemiological cohort in Iceland [35]. The current work extends this analysis to evaluate the association between these CT-derived measures and prevalent vertebral fractures. The same cohort, measurement techniques, and statistical methods were employed. As a result, the Materials and Methods section partially overlaps with the earlier publication; however, this is the first study to systematically compare the relevance of muscle parameters for both the association with and prediction of prevalent and incident vertebral fractures.

## Methods

As already stated in the Introduction, results of this study are based on the same cohort and CT scans as in the previously published analysis of incident fractures [34].

### Subjects

This study is based on a retrospective evaluation of CT images of the lumbar vertebrae L1 and L2 obtained from a subset of more than 5,000 older adults in Iceland who were enrolled in the Age, Gene/Environment Susceptibility–Reykjavik (AGES-Reykjavik) epidemiological study [35]. In a previous investigation, a case–control approach was used to select 843 participants (497 women and 346 men) from this cohort [36]. Briefly, participants were selected using a nested case-cohort design, including all participants with incident vertebral fractures within five years and a random subcohort of participants without incident vertebral fractures within this period .Individuals were excluded if they had a history of bone-active medications or metabolic bone diseases such as Paget’s disease, primary hyperparathyroidism, or bone metastases.

The present analysis utilized baseline data only. Prevalent vertebral fractures (VF), defined as fractures present at the time of the baseline examination, were identified using CT scout images acquired during the initial CT scan. Vertebral fractures were classified according to the Genant semi-quantitative (SQ) grading system [37]. The scout scans covered T6-L4. “First, the 5-year follow‐up CT scout for all participants were examined….Then, if a fracture was found in any vertebra from T6 to L4, the baseline CT scout was read to classify the fracture as prevalent or incident” [36].

In an additional analysis, individuals who developed a second vertebral fracture within five years were excluded, and baseline data alone were again analyzed.

### CT scanning and analysis

**Fig. 1:**
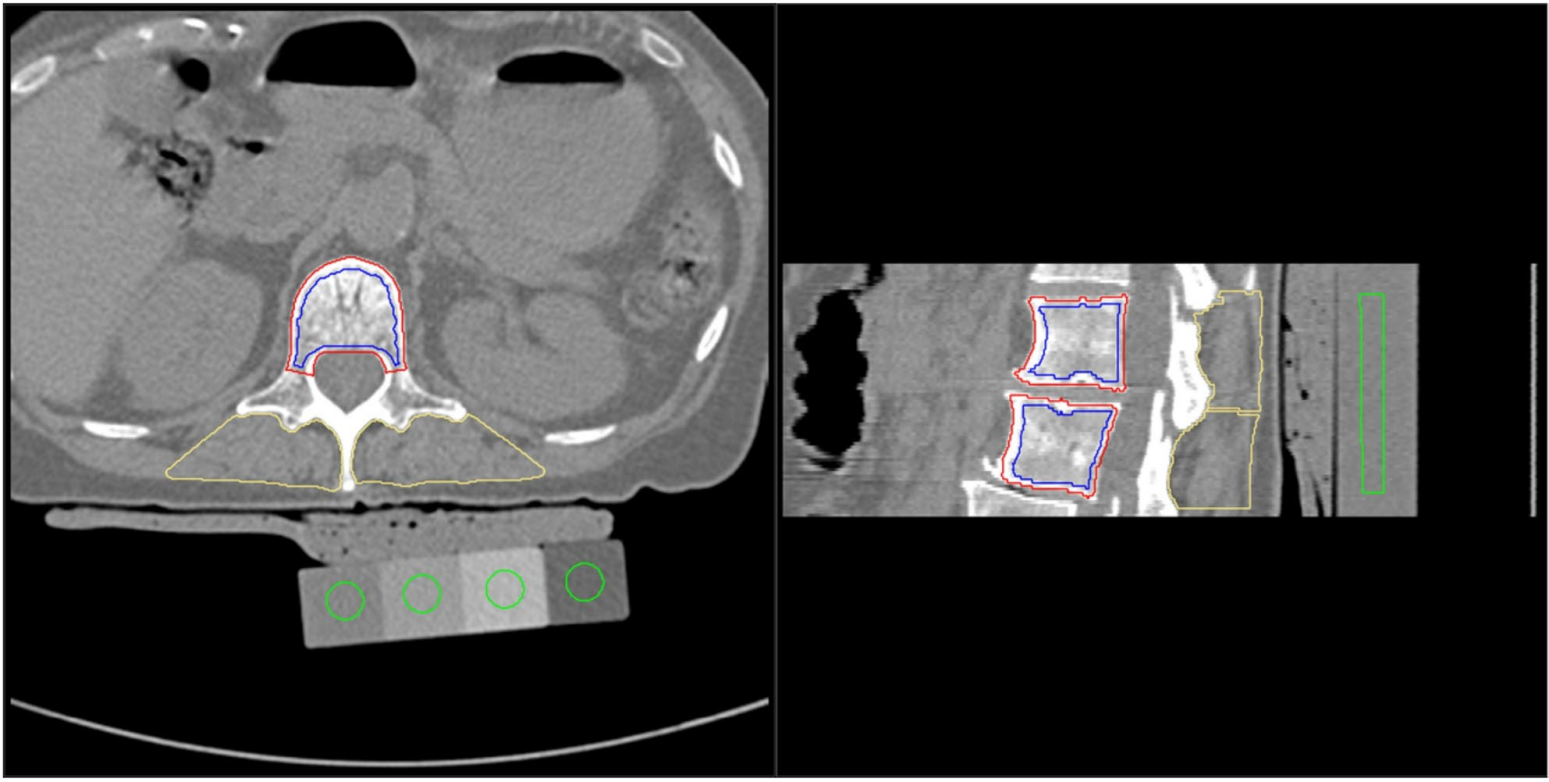
CT of the lumbar spine covering L1 and L2. The images show cropped axial and sagittal views. The green cylinders show the volumes of interest (VOIs) used to analyze the 4 different density compartments of the Image Analysis type 4 phantom. The red and blue contours delineate the integral and trabecular VOIs resulting from the 3D segmentation of L1 and L2. The yellow contours delineate the autochthonous muscle VOIs for L1 and L2

All CT examinations were conducted using a single scanner model (Sensation 4, Siemens, Erlangen, Germany) and identical acquisition and reconstruction parameters, including a tube voltage of 120 kV, tube current modulation with an effective dose of 150 mAs, a 50 cm field of view, a slice thickness and reconstruction increment of 1 mm, and a B30s reconstruction kernel. Simultaneous calibration of CT-derived attenuation values to bone mineral density was performed using a Type 4 Image Analysis Phantom (Image Analysis, Inc., Columbia, KY). In the majority of participants, the scan covered the L1 and L2 vertebral bodies. When either vertebra was excluded from analysis due to fracture or other abnormalities, alternative adjacent levels (T12 and L1 or L2 and L3) were evaluated instead. Quantitative computed tomography (QCT) analysis was performed using MIAF-Spine (Medical Image Analysis Framework, University of Erlangen) software version 6.0.7 (Fig. 1). For each subject, all QCT-derived measurements were averaged across the two vertebrae included in the scan. It should be noted that DXA measurements were not available for this study.

A comprehensive analysis was performed encompassing three distinct QCT parameter subsets (S1–S3). The first subset (S1, BMD set) included standard measurements of integral, cortical, and trabecular bone mineral density, bone mineral content, vertebral volume, and cortical thickness [38]. The second subset (S2, texture set) comprised parameters describing the trabecular microarchitecture of the vertebral body. The third subset (S3, muscle set) consisted of parameters characterizing the autochthonous paraspinal muscles at the vertebral levels imaged (Fig. 1). These muscles were analyzed as a single group, without further subdivision, and the psoas muscle was not included. To enhance measurement reproducibility, the peripheral regions of the autochthonous muscles were excluded during segmentation. Muscle fat infiltration patterns were subsequently quantified using texture-based parameters. A full description of all 50 parameters analyzed and additional methodological details are provided in the supplementary material.

### Statistics

The initial dataset comprised age, BMI and 50 variables analyzed by MIAF-Spine. All 50 variables were used to discriminate between prevalent VF. Specifically, S1 comprised 18 variables, S2 comprised seven variables, and S3 comprised 25 variables. Detailed descriptions can be found in the supplement. Z-transformation was employed to standardize all variables. Subsequent analyses were conducted in two distinct groups: men and women. Sex-specific standard deviations of the control group were utilized for standardization purposes. Standardized, age- and BMI-adjusted, univariate odds ratios (OR) were calculated for each parameter.

Stepwise logistic regression was used to obtain multivariable fracture discrimination models for each subset (S1–S3), with variable selection guided by the Akaike information criterion [39]. Bidirectional stepwise selection was initiated with a model comprising only age and BMI, and the inclusion or exclusion of variables was iteratively evaluated. In the final results, age and BMI were retained in all models, irrespective of their statistical significance. Other non-significant variables (*p* > 0.05) were excluded. The variance inflation factor (VIF) was employed to assess multicollinearity. Variables with VIF values greater than 5 were systematically eliminated, beginning with those exhibiting the highest VIF. Following each elimination, the model was re-evaluated, resulting in the exclusion of further non-significant variables. This iterative process was repeated until all VIF values were below 5, thereby ensuring minimal collinearity among the final variables in each model subset.

The BMD model S1 was selected as the reference model. Significant variables from another subset model (e.g. the muscle model) were integrated into the S1 model to create combined models. The ability of these combined models to discriminate prevalent fractures was then compared with that of S1 using likelihood ratio tests (LRT) between nested logistic models, in line with the approach suggested by Harrell [39]. The LRT adheres to a chi-squared distribution and provides p-values for comparing nested models. Receiver operating characteristic (ROC) curves and their area under the curve (AUC) values were also used as performance metrics and were compared using bootstrap confidence intervals (CIs) and tests [40].

The same procedure was used to compare the fracture discrimination of subsets S1–S3 with that of age and BMI-based discrimination. To assess the stability of the stepwise model selection process, a bootstrap analysis was performed using 1,000 resampled datasets. For each bootstrap sample, the stepwise procedure was repeated and the frequency with which the discriminator was included in the resulting models was recorded. Sign consistency reflects the proportion of bootstrap samples in which the estimated coefficient for a predictor retains the same direction (positive or negative), indicating stability in the sign of effect across resampled datasets. Additionally, area under the curve (AUC) values were calculated for each iteration to evaluate variability in model performance.

Furthermore, combined models were also calculated for women with fracture SQ grades of 2 and 3, thereby excluding mild SQ 1 fractures. Finally, the analysis was repeated for subgroups of women and men who did not experience an incident fracture within five years of the initial CT scan.

All statistical analyses were performed using R (R Core Team, version 4.3.2) and the functions ‘stepAIC’ (package: MASS) and ‘roc.test’ (package: pROC).

## Results

**Fig. 2:**
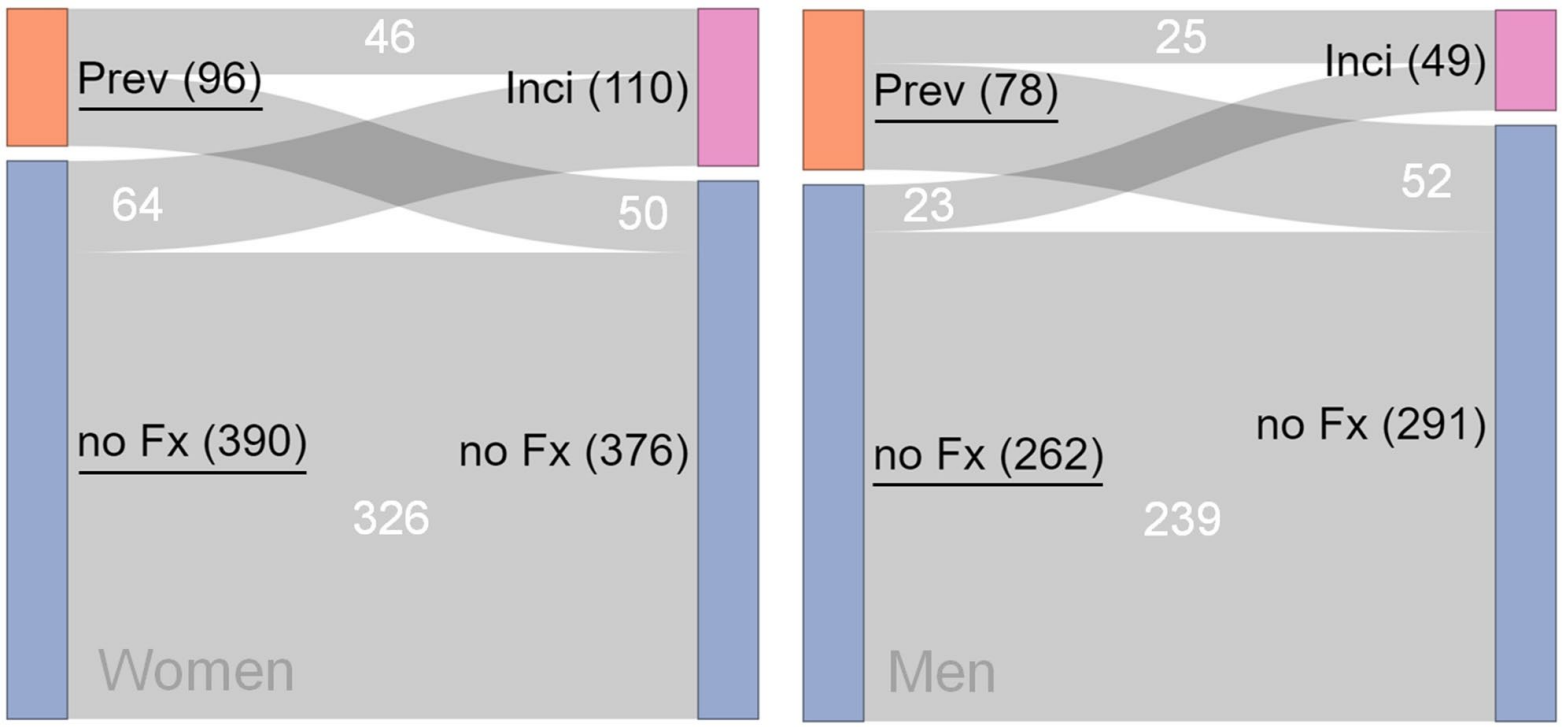
Sankey plots illustrating the populations of female and male subjects at baseline (left side) and follow-up (right side), with prevalent (Prev), incident (Inci), and no vertebral fractures (no Fx). Black numbers indicate the number of patients in each bar, while white numbers denote the contributions from other bars. In this study QCT images corresponding to the left (underlined) blue and orange bars were analyzed

A total of 826 CT datasets (486 women and 340 men) from the original subsample of 843 participants were analyzed. Analysis of CT scans from 17 subjects was not possible, primarily due to excessive osteophyte formation and substantial bone sclerosis around the endplates. At baseline, 96 of the 486 women and 78 of the 340 men had a VF (Fig. 2). Of these, 46 women and 26 men experienced a second VF within five years of the baseline assessment. Among women, 42 prevalent VFs were categorized as SQ 1, while an additional 54 were categorized as SQ 2 or SQ 3.

Patient characteristics and significant univariate discriminators for fracture occurrence in women and men are shown in Tables 1 and 2, respectively. All univariate ORs were adjusted for age and BMI. In women, 23 of the initial 50 variables were found to be significant discriminators of prevalent fractures, whereas in men, only five were significant. The non-significant variables (*p* > 0.05) are not displayed in the tables.

**Table 1:** Mean ± SD of univariate discriminators with area under curve (AUC) values and their confidence intervals (CI) for women; all values are sorted by Odds ratios (OR). OR are calculated per one standard deviation decrease. AUC and OR values are adjusted for age and BMI, n.s. discriminators are not listed here. CI gives the confidence interval of OR and p the significance level. A detailed description of the parameters is given in the Supplement

		Controls	Prevalent VF					
	*n*	326	96					
		Mean ± SD		AUC	CI	OR/SD	CI	*p*
	Age [y]	74.1 ± 5.1	77.3 ± 5.1	0.58	(0.51 ; 0.64)	0.55	(0.4 ; 0.7)	< 0.001
	BMI [kg/m^2^]	27.6 ± 4.6	26.5 ± 4.2	0.68	(0.62 ; 0.74)	1.31	(1.0 ; 1.7)	< 0.05

Subset	Discriminator	Mean ± SD				OR/SD	CI	*P*
S1-BMD	BMD_Trab_cCy [mg/cm^3^]	75.6 ± 29.3	49.5 ± 21.6	0.78	(0.72 ; 0.83)	3.32	(2.3 ; 5.0)	< 0.001
S1-BMD	BMD_Trab_mCy [mg/cm^3^]	81.0 ± 32.9	51.9 ± 26.1	0.77	(0.72 ; 0.82)	3.05	(2.1 ; 4.5)	< 0.001
S1-BMD	BMD_Trab_tVB [mg/cm^3^]	88.4 ± 30.7	62.5 ± 21.5	0.77	(0.72 ; 0.82)	2.98	(2.1 ; 4.4)	< 0.001
S1-BMD	BMD_Int_tVB [mg/cm^3^]	172.5 ± 37.6	144.0 ± 26.2	0.75	(0.70 ; 0.80)	2.42	(1.8 ; 3.4)	< 0.001
S1-BMD	BMC_Trab_tVB [g]	1.68 ± 0.56	1.28 ± 0.45	0.74	(0.69 ; 0.79)	2.14	(1.6 ; 3.0)	< 0.001
S1-BMD	BMD_Cort_tVB [mg/cm^3^]	363.1 ± 53.3	329.7 ± 40.4	0.73	(0.67 ; 0.78)	1.84	(1.4 ; 2.5)	< 0.001
S3-Muscle	%MT_Bin3	9.0 ± 2.9	10.4 ± 3.0	0.73	(0.68 ; 0.79)	0.56	(0.4 ; 0.7)	< 0.001
S3-Muscle	%MT_Bin4	17.3 ± 3.6	18.7 ± 3.3	0.72	(0.66 ; 0.78)	0.59	(0.4 ; 0.8)	< 0.001
S3-Muscle	%MT_Bin6	32.7 ± 7.5	28.2 ± 7.0	0.73	(0.68 ; 0.78)	1.7	(1.3 ; 2.2)	< 0.001
S3-Muscle	M_Diff_Box_Bin6	2.32 ± 0.14	2.24 ± 0.14	0.73	(0.68 ; 0.79)	1.67	(1.3 ; 2.1)	< 0.001
S3-Muscle	%MT_avg	75.5 ± 6.6	72.3 ± 7.2	0.72	(0.67 ; 0.78)	1.65	(1.3 ; 2.1)	< 0.001
S1-BMD	BMD_Cort_mVB [mg/cm^3^]	398.8 ± 63.3	362.6 ± 52.0	0.71	(0.65 ; 0.79)	1.59	(1.2 ; 2.0)	0.001
S3-Muscle	M_HU_avg	11.4 ± 11.6	5.2 ± 12.8	0.72	(0.67 ; 0.78)	1.58	(1.2 ; 2.0)	< 0.001
S3-Muscle	%MT_Bin5	31.4 ± 4.6	31.1 ± 4.7	0.70	(0.64 ; 0.76)	1.53	(1.1 ; 2.1)	0.01
S1-BMD	BMC_Int_tVB [g]	5.50 ± 1.2	4.88 ± 1.1	0.71	(0.65 ; 0.76)	1.51	(1.2 ; 2.2)	0.004
S3-Muscle	M_Vol [cm^3^]	114.3 ± 19.7	104.0 ± 21.9	0.71	(0.65 ; 0.76)	1.51	(1.2 ; 1.9)	< 0.001
S3-Muscle	%MT_Bin2	5.7 ± 2.3	7.1 ± 2.9	0.71	(0.66 ; 0.77)	0.67	(0.5 ; 0.8)	< 0.001
S1-BMD	Vol_Int_tVB [cm^3^]	32.0 ± 3.9	34.0 ± 4.9	0.70	(0.64 ; 0.76)	0.68	(0.5 ; 0.8)	< 0.001
S1-BMD	Vol_Trab_tVB [cm^3^]	19.3 ± 2.8	20.7 ± 3.4	0.70	(0.64 ; 0.76)	0.68	(0.5 ; 0.9)	< 0.001
S2-Texture	Trab_Entropy	5.23 ± 0.23	5.13 ± 0.3	0.70	(0.63 ; 0.76)	1.40	(1.1 ; 1.7)	< 0.01
S1-BMD	Thick_Cort_mVB [mm]	0.98 ± 0.28	0.89 ± 0.23	0.70	(0.64 ; 0.76)	1.35	(1.0 ; 1.8)	0.03
S3-Muscle	M_Entropy_Bin6	4.54 ± 0.56	4.67 ± 0.6	0.69	(0.63 ; 0.75)	0.73	(0.6 ; 0.9)	0.02
S1-BMD	Vol_Cort_tVB [cm^3^]	7.6 ± 1.2	7.8 ± 1.3	0.69	(0.63 ; 0.75)	0.77	(0.6 ; 1.0)	0.02

**Table 2:** Mean ± SD of univariate discriminators with area under curve (AUC) values and their confidence intervals (CI) for men; all values are sorted by Odds ratios (OR). OR are calculated per one standard deviation decrease. AUC and OR values are adjusted for age and BMI, not significant discriminators are not listed here. CI gives the confidence interval of OR and p the significance level. A detailed description of the parameters is given in the Supplement

		Controls	Prevalent VF					
	*n*	239	78					
		Mean ± SD		AUC	CI	OR/SD	CI	*p*
	Age [y]	74.6 ± 5.1	76.2 ± 5.3	0.59	(0.52 ; 0.66)	0.74	(0.6 ; 1.0)	0.02
	BMI [kg/m^2^]	26.8 ± 3.6	27.0 ± 4.1	0.52	(0.44 ; 0.60)	0.94	(0.7 ; 1.2)	0.65

Subset	Discriminator	Mean ± SD				OR/SD	CI	*p*
S1-BMD	Vol_Int_tVB [cm^3^]	43.2 ± 5.6	45.4 ± 6.6	0.64	(0.57 ; 0.71)	0.70	(0.5 ; 0.9)	0.004
S1-BMD	Vol_Trab_tVB [cm^3^]	26.8 ± 4.0	28.4 ± 4.9	0.64	(0.57 ; 0.71)	0.70	(0.5 ; 0.9)	0.003
S1-BMD	BMD_Cort_tVB [mg/cm^3^]	408.4 ± 60.0	390.1 ± 67.5	0.63	(0.56 ; 0.70)	1.38	(1.1 ; 1.8)	0.02
S1-BMD	BMD_Int_tVB [mg/cm^3^]	193.3 ± 39.7	181.2 ± 41.8	0.63	(0.56 ; 0.70)	1.37	(1.0 ; 1.8)	0.02
S1-BMD	BMD_Trab_mCy [mg/cm^3^]	91.4 ± 34.5	80.6 ± 34.4	0.63	(0.56 ; 0.70)	1.35	(1.0 ; 1.8)	0.04

Among women a large number of bone and muscle parameters significantly discriminated between prevalent VF. A detailed description of the parameters is provided in the Supplement. Trabecular BMD parameters showed ORs of at least 3, whereas the ORs for muscle parameters were below 2. Trabecular entropy was the only trabecular texture parameter that significantly discriminated prevalent VF. In men, only bone parameters discriminated prevalent VF. The ORs were smaller than those in women. Neither trabecular texture parameters nor muscle parameters significantly discriminated against prevalent VF. The AUCs were almost identical for all five parameters in men (0.63). In women, AUC values ranged from 0.69 for cortical volume of the total vertebral body to 0.78 for trabecular BMD of the central cylinder, although the confidence intervals largely overlapped. Interestingly, both women and men in the fractured group had higher vertebral body volume than those in the control group.

S1, S2 and S3 present the results of the subset-specific stepwise logistic regressions. The first shows the AUC for the combination of just age and BMI, then the AUCs for the models S1-S3 and finally the combined models in the last two rows. The predictors that remained in the models S1-S3 are listed in Tables S2 and S3. Table 3 shows the performance of combinations of nested models.

**Table 3:** AUC values for a combination of age and BMI and for the subset specific models (that are also adjusted for age and BMI)

	Women				Men		
	AUC	CI	*p* compared to Age & BMI	*p* compared to S1 BMD	AUC	CI	*p* compared to Age & BMI
Age & BMI	0.69	(0.62 ; 0.74)	-	-	0.60	(0.53 ; 0.67)	-
S1 BMD	0.80	(0.75 ; 0.86)	< 0.001	-	0.64	(0.57 ; 0.71)	n.s.
S2 Texture	0.70	(0.63 ; 0.76)	n.s.	-	*		
S3 Muscle	0.77	(0.72 ; 0.82)	< 0.001	-	*		
S1 BMD & S2 Texture	0.81	(0.76 ; 0.86)	-	n.s.	*		
S1 BMD & S3 Muscle	0.84	(0.79 ; 0.88)	-	< 0.01	*		

*n.s* Not significant

*No predictors of S2-Texture or S3-Muscle remained in the final model

For women four bone parameters, one texture parameter, and three muscle parameters remained significant discriminators in the corresponding models after logistic regression (Table S2). The LRT results (Table 4) show that models S1, S2, and S3 exhibited significantly higher fracture discrimination (*p* ≤ 0.01) than the combination of age and BMI alone. Furthermore, the combination of BMD with texture and muscle models showed significantly better fracture discrimination than BMD alone. In the combined S1–S2 model, all parameters from the S1 and S2 models remained significant (Table S2). In the combined S1–S3 model, Thick_Cort_lE and M_Entropy_Bin6 were no longer significant.

**Table 4:** Performance of combinations of nested models tested by LRT: Model 1, which is the base model, and Model 2, which represents the combined model

Comparison of Nested Models		Women			Men		
Model 1	Model 2	DoF	χ2	*p*	DoF	χ2	*p*
Age & BMI	S1 BMD	5	72.6	<0.001	1	8.9	0.003
Age & BMI	S2 Texture	1	10.4	<0.001	*		
Age & BMI	S3 Muscle	3	36.5	<0.01	*		
S1 BMD	S2 Texture	1	6.2	0.01	*		
S1 BMD	S3 Muscle	3	28.7	<0.001	*		

*No predictors of S2-Texture or S3-Muscle remained in the final models

The AUC values for S1 and the S1-S3 combination were significantly higher than for the combination of age and BMI, but the S1-S2 combination was not (Table 3). The AUC values for the S1-S3 combination were also significantly higher than those for the combination of age and BMI.

In men the results were different. All texture and muscle parameters were removed by stepwise logistic regression. In addition to age and BMI, only one bone parameter – the trabecular volume of the total vertebral body (Vol_Trab_tVB) - remained a significant discriminator of prevalent VF (Table S3). The LRT results show that the S1 model was superior to the combination of age and BMI for fracture discrimination (Table 4). However, the AUC values did not differ significantly between the L1 model and the combination of age and BMI.

The bootstrap procedure showed that, when age and BMI were included, an average of 8.42 (CI 5–12) predictors remained significant for women, with a mean area under the curve (AUC) of 0.83 (CI 0.77–0.88). Among men, an average of 6.23 (CI 3–11) predictors remained significant, with a mean AUC of 0.71 (CI 0.63–0.81). Table S4 documents the frequency of significant predictors in each of the 1,000 resampled datasets.

For comparison purposes, the AUC values were also calculated for a model selected based on clinical expertise. In addition to age and BMI, this model included integral BMD of the total vertebral body, the standard QCT parameter, and cortical thickness measured at the midsection of the vertebral body, a parameter representing the geometry of the vertebral body. In both women and men, cortical thickness was not a significant discriminator in the models. The AUC results were 0.75 (95% CI 0.70–0.80) for women and 0.63 (95% CI 0.56–0.71) for men.

Among women with prevalent VF of grades SQ 2 or SQ 3 (excluding SQ 1 VF), the AUC values (Table S5) increased to 0.83 (95% CI 0.78–0.89), 0.74 (95% CI 0.67–0.81) and 0.85 (95% CI 0.80–0.90) for S1, S2 and S3, respectively, compared to the values in Table 3. The performance of the combined models is shown in Table S6. Compared to S1 alone, the combination of S1 and S2 showed a borderline significant increase in fracture discrimination (*p* = 0.05), which was not observed in the subset encompassing fractures of grades SQ1 to SQ3. There were too few cases of men to perform such an analysis.

Of the 96 women and 78 men with prevalent fractures, 46 and 25, respectively, experienced a second VF within 5 years. Tables S7 and S8 show equivalent AUC results to those in Tables 3 and 4 when subjects with a second fracture are excluded. No statistical comparisons were performed between the full group and the subgroup of subjects, but the trends were comparable. In women, the S1, S2 and S3 models showed better discrimination for prevalent VFs than age combined with BMI, and S2 or S3 improved discrimination significantly compared to S1 (Table S6). AUC values significantly improved for the nested S1 and S3 models (Table S5). In men, even the S1 model did not improve the discriminative performance of age and BMI.

## Discussion

This retrospective analysis of the AGES-Reykjavik study revealed that prevalent vertebral fractures in women are associated with parameters characteristic of autochthonous muscles, but not in men. In women with prevalent vertebral fractures (VF) compared to those without, the CT value, commonly referred to as muscle density, and the percentage of muscle tissue were lower, and muscle volume was smaller, after adjusting for age and BMI. These results are consistent with those of a recent meta-analysis [31]. The significance of muscle parameters remained after further adjustment for standard QCT parameters, such as BMD and BMC, as well as the volume and cortical thickness of the L1 and L2 vertebrae.

Furthermore, multivariable statistics revealed that, in women, combining the S1 BMD model with the S3 muscle model significantly improved VF discrimination compared to using the BMD model alone. Excluding mild VF (SQ1) resulted in a further increase in the AUC value of the combined S1-S3 model, from 0.84 to 0.89. The results were consistent regardless of whether the women experienced a second VF within five years.

These findings differ from those reported in a recent publication on the prediction of first-time VFs using spinal QCT in the same cohort [34]. In women, no muscle parameter was found to be predictive, even before adjustment for BMD parameters. Therefore, results from cross-sectional studies, which differentiate existing VF, cannot be generalized to prospective studies of incident VF and are of limited value in determining CT risk factors. However, cross-sectional studies seem useful for investigating muscle damage after VF. There is evidence that paraspinal muscle deterioration follows VF [24, 30, 32, 41, 42] as well as evidence of progressive vertebral collapse [43]. Therefore, once muscle damage has occurred, the risk of further VFs seem to increase. It could be speculated that muscle deterioration is usually not present prior to the first VF but obviously further studies are required.

However, findings were very different in men: muscle or texture parameters did not contribute to the discrimination of VF, even before adjustment of BMD. Nevertheless, there was a significant, albeit clinically irrelevant, contribution of muscle parameters to the prediction of the first incident VF [34].

The results of the AGES-Reykjavik study with respect to the relevance of muscle properties in a Caucasian population are unique. To date, only one other longitudinal study has examined CT-based bone and muscle parameters to predict the risk of a first incident VF. This study, conducted in a Chinese population, reported significant risk prediction for paraspinal CT density and area in women, and for muscle area in men, when adjusted for age and BMI [22, 33]. However, an adjustment for vertebral body BMD was not performed, so it remains unclear whether combining muscle parameters with BMD parameters further increases risk prediction.

Most cross-sectional studies investigating the association between muscle parameters and vertebral fractures did not report sex-specific data. The results are often biased by the fact that more women than men are included. To fully understand the differences in fracture discrimination and prediction, it is recommended that results are reported separately for men and women, rather than simply applying an adjustment for sex. Sex-specific differences in bone and muscle properties are well documented. For instance, men have greater vertebral strength at all ages, primarily due to their larger cross-sectional area (CSA) [27, 44, 45]. All studies on VF discrimination or prediction that performed a sex-specific analysis, reported sex-specific differences [27, 28, 34, 46].

In this study, the BMD model S1, which was extracted from 18 different BMD and cortical thickness measures, was used as the reference model because these parameters are commonly obtained in QCT and can be measured with good precision [38]. Age and BMI alone significantly discriminated VF with AUC values of 0.69 in women and 0.6 in men. Therefore, to be consistent with most fracture discrimination publications that often report age- and BMI-adjusted odds ratios, age and BMI were retained in all models, even if they were not significant in the regression.

Following variable reduction in the stepwise logistic regression, several parameters remained in the S1 model for women, but only trabecular volume remained for men. The sign consistency for trabecular volume in men was low in the bootstrap analysis. In approximately half of the 1000 models, prevalent VFs were associated with higher trabecular volume, while in the other models, they were associated with lower trabecular volume.

The bootstrap analysis showed that there is no optimal set of variables for fracture discrimination. Even a small variation in the study population will result in different variable selection in the stepwise regression. For women, the AUC of 0.8 for the S1 model was very close to the average AUC of 0.83 for the 1000 bootstrap models. For men, however, the AUC of 0.64 was close to the lower confidence interval of the AUC. None of the 18 input BMD parameters used to construct the S1 model for males occurred in more than 50% of the 1000 S1 models. It is unlikely that the S1 reference models in this study overestimated fracture discrimination performance due to overfitting, a common problem in multivariable analyses. There is no single best set of S1 QCT variables for fracture discrimination. Conducting a separate analysis of a distinct subset of the AGES population, or even of a different study population, is likely to produce a different S1 reference model. This phenomenon is also evident in the univariate results, where the adjusted odds ratios (ORs) for many variables were found to be highly comparable despite adjustments for age and body mass index (BMI).

The bootstrap analysis was only performed for the S1 model. Similar outcomes can be expected for the S2 and S3 models. This also explains why, in the cross-sectional studies cited in the introduction, a wide range of different muscle parameters were associated with VF.

In particular in radiomics techniques, for example the analysis of trabecular texture, overfitting is a common problem that frequently results in very high area under the curve (AUC) values of greater than 0.9, or even 0.95. In the analysis of the AGES-Reykjavik study, parameters describing trabecular texture improved the prediction of the first incident VF compared to BMD parameters alone [34], but did not improve the discrimination of prevalent VF. We applied likelihood ratio tests (LRT) between nested logistic regression models to determine whether texture parameters added information to BMD parameters. Most other studies neither investigated the performance gain over BMD nor reported the effect sizes of radiomics features in regression models that also included BMD [47–49].

One limitation of the study is the fact that only L1 and L2 were analyzed. This is the standard in QCT [50] but the analysis of other vertebrae may have changed the results. Another limitation is the use of CT scout views instead of X-rays to identify and grade the vertebral fractures. Also the scout scans did not include the upper thoracic vertebrae. Further, due to the limited spatial resolution of the CT scans, cortical thickness was overestimated. A more accurate analysis [51, 52] may have retained cortical thickness in the final models.

## Conclusion

In Caucasian women, but not men, muscle parameters improve discrimination of prevalent vertebral fracture compared to BMD parameters alone, but muscle parameters are not relevant for predicting first incident VF [34]. Other studies have shown that muscle deterioration occurs after the first VF and that muscle parameters improve the prediction of a second VF. It is not possible to identify a unique combination of parameters, with or without the contribution of muscle parameters, that provides the best fracture discrimination and prediction. Therefore, easy-to-measure QCT parameters with evidence of good precision may be preferable to more esoteric parameters, although, after adjusting for age and BMI, some of these will also provide acceptable VF discrimination and prediction but will not usually outperform BMD parameters.

## Supplementary Information

Supplementary Material 1

## Data Availability

Data are principally available through the Icelandic Heart Association (hjarta.is). Please check for terms and conditions with the IHA.

## Abbreviations

AUC: Area under the curve
BMC: Bone mineral content
BMD: Bone mineral density
BMI: Body mass index
CI: Confidence interval
CSA: Cross-sectional area
CT: Computed tomography
DXA: Dual-energy X-ray absorptiometry
FEA: Finite element analysis
LRT: Likelihood ratio test
MRI: Magnetic resonance imaging
QCT: Quantitative computed tomography
OR: Odds ratio
ROC: Receiver operating characteristic
S1: BMD set - included standard measurements of integral, cortical, and trabecular BMD, BMC, vertebral volume, and cortical thickness
S2: Texture set – included parameters describing the trabecular microarchitecture of the vertebral body
S3: Muscle set – included parameters characterizing the autochthonous paraspinal muscles
SQ: Genant semi-quantitative grading system for vertebral fractures
VF: Vertebral fractures
VIF: Variance inflation factor
Vol: Volume
