# Supplementary material for: Association of trabecular texture and paraspinal muscle characteristics with prevalent vertebral fractures - QCT results from a subcohort of the AGES population
Source: BMC Musculoskelet Disord. 2026 Jun 27;27:558. doi: 10.1186/s12891-026-09893-9 (PMC13321617; doi:10.1186/s12891-026-09893-9)
Supplement: Supplementary file 1 — Supplementary Material 1 [file 12891_2026_9893_MOESM1_ESM.docx]

Association of Trabecular Texture and Paraspinal Muscle Characteristics with Prevalent Vertebral Fractures - QCT Results from a Subcohort of the AGES Population - Supplement

# Variables used for prevalent vertebral fracture discrimination

Apart from age and BMI all variables were divided into three subsets containing discriminators related to BMD, trabecular bone texture, and autochthonous muscle

## BMD subset

The BMD subset included BMD, BMC and Volume assessments of various bone compartments and cortical thickness as shown in Table S1, VOIs are visualized in Figure S1. In the paper predictor names followed the following conventions: The measured value was appended by the abbreviations of the bone compartment and the volume of interest, e.g. BMD_Int_tVB, or Vol_Cort_mVB, or Thick_Cort_uE

## Trabecular texture subset

The following texture parameters were determined from the distribution of the CT values in the trabecular compartment of the total vertebral body [1]

1. Global inhomogeneity (Trab_gInhomo)
2. Local inhomogeneity (Trab_lInhomo)
3. Global anisotropy (Trab_gAniso)
4. Local anisotropy (Trab_lAniso)
5. Variogram slope (Trab_Vario_slop)
6. Entropy (Trab_Entrop)
7. Differential Box Counting (Trab_Diff_Box)

This specific selection was mostly made from the perspective of medical interpretability. Inhomogeneity is the same as BMD standard deviation, anisotropy characterizes the directedness of the trabeculae an important feature as most trabecular in the lumbar vertebrae are aligned in horizontal or vertical direction, The variogram slope is the basis of the so-called trabecular bone score (TBS) used in DXA to predict spinal fractures [2]. We investigated the dependence of the first seven parameters on noise and spatial resolution previously [3, 4]. Differential box counting, was added as a fractal measure.

## Muscle subset

The following parameters were determined from the segmented combined left and right autochthonous muscles.

1. Muscle volume (M_Vol)
2. Muscle density (M_HU) determined as average CT value
3. Percent muscle tissue (%MT) determined as percent of volume with a CT value > 48 HU [5]

Then a histogram analysis of the CT values of the segmented autochthonous muscles was performed to divide the muscle into 6 partitions (Bins): Bin 1: all voxels of pure adipose tissue and Bin 6: all voxels of pure muscle tissue (MT)(defined as HU > 48). Bins 2, 3, 4 and 5 consisted of voxels representing up to 25%, 50%, 75% and 100% MT, accordingly (Figure S2). In order to assess the adipose tissue distribution of the autochthonous muscles, the following variables were used:

1. Percent muscle tissue (%MT) in each bin denoted as %MT_Bin1 to %MT_Bin6
2. Mean HU value of Bin 1 and Bin 6 (Mus_HU_Bin1, Mus_HU_Bin6)
3. The following texture parameters determined for Bin 1 and Bin 6
   1. Global inhomogeneity (M_gInhomo_Bin1, M_gInhomo_Bin6)
   2. Local inhomogeneity (M_lInhomo_Bin1, M_lInhomo_Bin6)
   3. Global anisotropy (M_gAniso_Bin1, M_gAniso_Bin6)
   4. Local anisotropy (M_lAniso_Bin1, M_lAniso_Bin6)
   5. Variogram slope (M_Vario_slop_Bin1, M_Vario_slop_Bin6)
   6. Entropy (M_Entrop_Bin1; M_Entrop_Bin6)
   7. M_Differential Box Counting (M_Diff_Box_Bin1, M_Diff_Box_Bin6)

# Supplementary Figures

Figure S1: An overview of the different volumes of interest (VOI) used to measure BMD. The measures are shown differently for L1 and L2, but they are applicable to both vertebrae. The total vertebral body VOI (tVB) is shown in red (with the trabecular VOI in dark blue). The central cylinder (cCy) VOI and its mid-section (mCy) are shown in cyan in L2, in both sagittal and coronal views. In L1, the upper vertebral endplate (uE) VOI is shown in yellow, while the lower endplate (lE) VOI is shown in green. The mid-vertebral body VOI is defined as the volume between the upper and lower endplates (between the yellow and green borders), which is also shown in the sagittal and coronal views.


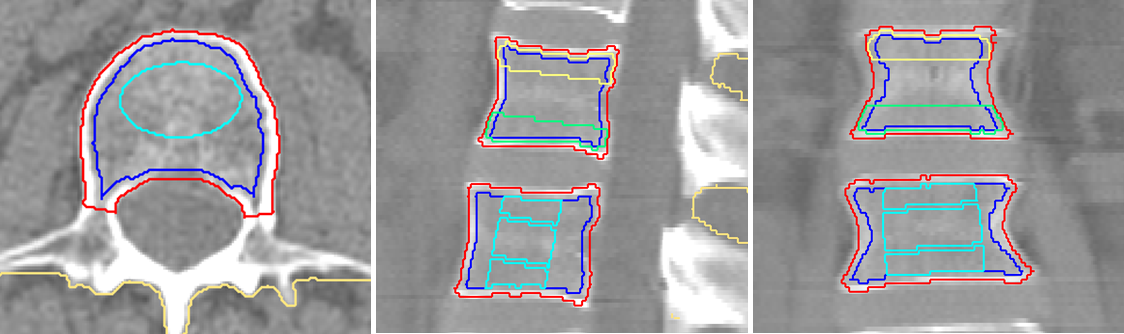


Figure S2: Left: segmentation of muscle VOI; right: histogram of CT values of muscle VOI and partition into 6 bins


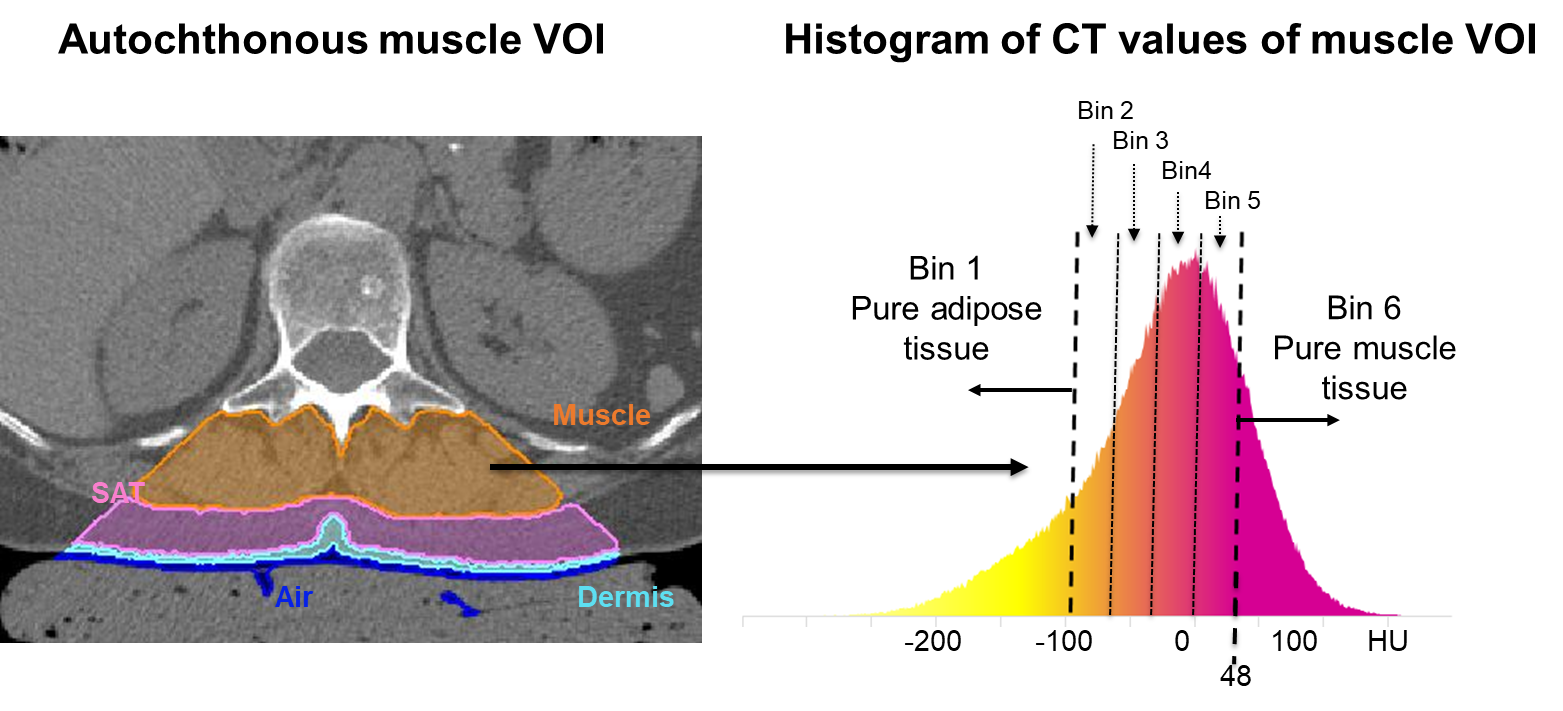


# Supplementary Tables

Table S1: Overview of BMD/BMC/Vol and cortical thickness assessments

|  | Total vertebral body (tVB) | | | | Central cylinder (cCy) | | Mid cylinder (mCy) | Mid vert body (mVB) | Upper endplate (uE) | | | Lower endplate (lE) |
| --- | --- | --- | --- | --- | --- | --- | --- | --- | --- | --- | --- | --- |
|  | Integral | Trabecular | Cortical | Trabecular | | Cortical | Trabecular | Cortical | | Cortical | Cortical | |
| BMD | X | X | X | X | | X |  | X | |  |  | |
| BMC | X | X | X | X | | X | X | X | |  |  | |
| Volume | X | X | X | X | | X |  | X | |  |  | |
| Thickness |  |  | X |  | |  |  | X | | X | X | |

Table S2: Women: Variables that remained in the individual models after binary logistic regression. Note: age and BMI were always retained in the models

|  | Beta | SE | | p |
| --- | --- | --- | --- | --- |
| **S1 BMD** |  | | | |
| Age | 0.086 | 0.194 | | 0.454 |
| BMI | -0.215 | 0.142 | | 0.131 |
| BMD_Trab_cCy | -0.913 | 0.207 | | <0.0001 |
| Vol_Cort_tVB | 1.008 | 0.211 | | <0.0001 |
| Thick_Cort_mVB | -0.759 | 0.222 | | <0.001 |
| Height | -0.399 | 0.143 | | <0.01 |
| Thick_Cort_lE | -0.320 | 0.155 | | 0.04 |
| **S2 Texture** |  | | | |
| Age | 0.580 | 0.118 | | <0.0001 |
| BMI | -0.182 | 0.128 | | 0.155 |
| Trab_Entropy | -0.334 | 0.103 | | <0.01 |
| **S3 Muscle** |  | | | |
| Age | 0.377 | 0.127 | 0.003 | |
| BMI | -0.617 | 0.155 | <0.0001 | |
| M_Diff_Box_Bin1 | 0.592 | 0.153 | <0.001 | |
| %MT_avg | 0.895 | 0.176 | <0.0001 | |
| M_Entropy_Bin6 | 0.286 | 0.131 | 0.03 | |

Table S3: Men: variables that remained in the individual models after binary logistic regression. Note: age and BMI were always retained in the models. There were no texture or muscle parameters that remained in the logistic regression models.

|  | Beta | SE | p |
| --- | --- | --- | --- |
| **S1 BMD** |  | | |
| Age (SD) | 0.35 | 0.13 | 0.008 |
| BMI (SD) | 0.09 | 0.13 | 0.48 |
| Vol_Trab_tVB | 0.36 | 0.12 | 0.003 |

Table S 4: Predictors sorted by frequency in percent that remained significant in the logistic regression models after resampling 1000 datasets in the bootstrap analysis. Sign consistency (in percent) indicates how often the regression coefficient (β) retained the same direction (positive or negative) across bootstrap samples. Predictors with lower sign consistency are considered unstable. Age and BMI always remained in the model regardless of their significance.

| **Women** | | | **Men** | | |
| --- | --- | --- | --- | --- | --- |
| **Variable** | **Frequency** | **Sign Consistency** | **Variable** | **Frequency** | **Sign Consistency** |
| AGE | 100 | 84.4 | AGE | 100 | 93.9 |
| BMI | 100 | 80.2 | BMI | 100 | 82.0 |
| Thick_Cort_mVB | 85.3 | 100 | Vol_Int_tVB | 45.2 | 94.9 |
| Height | 67.2 | 99.4 | Vol_Trab_tVB | 41.9 | 57.8 |
| Vol_Cort_tVB | 65.1 | 99.4 | BMD_Cort_tVB | 40.0 | 92.5 |
| Thick_Cort_lE | 64.4 | 100 | BMC_Int_tVB | 40.0 | 90.7 |
| BMD_Trab_cCy | 50.8 | 98.8 | Thick_Cort_tVB | 29.8 | 100 |
| Thick_Cort_uE | 35.2 | 92.6 | Thick_Cort_mVB | 24.6 | 99.6 |
| Vol_Cort_mVB | 34.4 | 95.3 | Thick_Cort_uE | 22.6 | 65.0 |
| Weight | 33.9 | 59.3 | Height | 22.2 | 90.5 |
| BMD_Trab_mCy | 27.1 | 62.7 | Vol_Cort_mVB | 21.4 | 88.3 |
| Vol_Int_tVB | 27.1 | 67.5 | Weight | 19.0 | 70.0 |
| Thick_Cort_tVB | 24.7 | 88.3 | BMD_Cort_mVB | 19.0 | 90.5 |
| Vol_Trab_tVB | 20.3 | 74.9 | BMC_Trab_tVB | 17.9 | 88.8 |
| BMC_Int_tVB | 19.9 | 97.5 | Vol_Cort_tVB | 17.1 | 92.4 |
| BMD_Cort_mVB | 19.2 | 97.9 | BMD_Trab_mCy | 16.6 | 92.8 |
| BMD_Trab_tVB | 17.2 | 58.1 | BMD_Trab_cCy | 15.7 | 97.5 |
| BMD_Cort_tVB | 12.6 | 79.4 | Thick_Cort_lE | 15.0 | 66.7 |
| BMC_Trab_tVB | 12.1 | 91.7 | BMD_Trab_tVB | 12.6 | 92.9 |
| BMC_Cort_tVB | 10.1 | 79.2 | BMC_Cort_mVB | 11.8 | 57.6 |
| BMC_Cort_mVB | 9.1 | 60.4 | BMD_Int_tVB | 11.7 | 63.2 |
| BMD_Int_tVB | 6.7 | 62.7 | BMC_Cort_tVB | 8.0 | 77.5 |

Table S 5: Subset of women with SQ2 and SQ3 fractures. AUC values for a combination of age and BMI and for the subset specific models (that are also adjusted for age and BMI). n.s.: not significant

|  | **Women** | | | |
| --- | --- | --- | --- | --- |
|  | **AUC** | **CI** | **p compared to Age & BMI** | **p compared to S1 BMD** |
| Age & BMI | 0.71 | (0.64 ; 0.78) |  |  |
| S1 BMD | 0.83 | (0.78 ; 0.89) | < 0.001 |  |
| S2 Texture | 0.70 | (0.63 ; 0.76) | n.s. |  |
| S3 Muscle | 0.77 | (0.72 ; 0.82) | < 0.001 |  |
| S1 BMD & S2 Texture | 0.84 | (0.79 ; 0.89) |  | n.s. |
| S1 BMD & S3 Muscle | 0.89 | (0.84 ; 0.93) |  | < 0.001 |

Table S 6: Subset of women with SQ2 and SQ3 fractures. Performance of combinations of nested models tested by LRT: Model 1, which is the base model, and Model 2, which represents the combined model.

| **Comparison of Nested Models** | | **Women** | | |
| --- | --- | --- | --- | --- |
| **Model 1** | **Model 2** | **DoF** | **LR χ2** | **p** |
| Age & BMI | S1 BMD | 5 | 49.4 | **<0.001** |
| S1 BMD | S2 Texture | 1 | 3.75 | **0.05** |
| S1 BMD | S3 Muscle | 3 | 34.3 | **<0.001** |

Table S 7: Subset of subjects without incident vertebral fracture. AUC values for a combination of age and BMI and for the subset specific models (that are also adjusted for age and BMI). *No predictors of S2-Texture or S3-Muscle remained in the final model. n.s.: not significant

|  | **Women** | | | | **Men** | | |
| --- | --- | --- | --- | --- | --- | --- | --- |
|  | **AUC** | **CI** | **p compared to Age & BMI** | **p compared to S1 BMD** | **AUC** | **CI** | **p compared to Age & BMI** |
| Age & BMI | 0.61 | (0.58 ; 0.76) |  |  | 0.61 | (0.53 ; 0.69) |  |
| S1 BMD | 0.78 | (0.71 ; 0.85) | < 0.01 |  | 0.63 | (0.55 ; 0.72) | n.s. |
| S2 Texture | 0.69 | (0.60 ; 0.77) | n.s. |  | * | | |
| S3 Muscle | 0.74 | (0.66 ; 0.81) | 0.03 |  | * | | |
| S1 BMD & S2 Texture | 0.79 | (0.72 ; 0.86) |  | n.s. | * | | |
| S1 BMD & S3 Muscle | 0.83 | (0.77 ; 0.89) |  | 0.02 | * | | |

Table S 8: Subset of subjects without incident vertebral fracture. Performance of combinations of nested models tested by LRT: Model 1, which is the base model, Model 2, which represents the combined model. * No predictors of S2-Texture or S3-Muscle remained in the final models.

| **Comparison of Nested Models** | | **Women** | | | **Men** | | |
| --- | --- | --- | --- | --- | --- | --- | --- |
| **Model 1** | **Model 2** | **DoF** | **χ2** | **p** | **DoF** | **χ2** | **p** |
| Age & BMI | S1 BMD | 2 | 31.3 | <0.001 | 1 | 2.2 | n.s. |
| Age & BMI | S2 Texture | 1 | 7.3 | <0.01 | * | | |
| Age & BMI | S3 Muscle | 3 | 14.6 | <0.01 | * | | |
| S1 BMD | S2 Texture | 1 | 4.4 | 0.04 | * | | |
| S1 BMD | S3 Muscle | 3 | 25.9 | <0.001 | * | | |

# References

1. Zerfass, P., Lowitz, T., Museyko, O., Bousson, V., Laouisset, L., Kalender, W.A., et al., *An Integrated Segmentation and Analysis Approach for QCT of the Knee to Determine Subchondral Bone Mineral Density and Texture.* IEEE Trans Biomed Eng, 2012. **59**(9): 2449-58.

2. McCloskey, E.V., Oden, A., Harvey, N.C., Leslie, W.D., Hans, D., Johansson, H., et al., *A Meta-Analysis of Trabecular Bone Score in Fracture Risk Prediction and Its Relationship to FRAX.* J Bone Miner Res, 2016. **31**(5): 940-8.

3. Lowitz, T., Museyko, O., Bousson, V., Kalender, W.A., Laredo, J.D., and Engelke, K., *Characterization of knee osteoarthritis-related changes in trabecular bone using texture parameters at various levels of spatial resolution-a simulation study.* Bonekey Rep, 2014. **3**: 615.

4. Lowitz, T., Museyko, O., Bousson, V., Kalender, W.A., Laredo, J.D., and Engelke, K., *A Digital Model to Simulate Effects of Bone Architecture Variations on Texture at Spatial Resolutions of CT, HR-pQCT, and muCT Scanners.* J Med Eng, 2014. **2014**: 946574.

5. Bammessel, J., Bartenschlager, S., Chaudry, O., Krekiehn, N., Johannesdottir, F., Wang, L., et al., *Quantification of Skeletal Muscle Density, Mass and Fat Fraction using Single-Energy Computed Tomography.* Journal Orthopedic Translations, 2026. **submitted**.
